# Application fruit tree hole storage brick fertilizer is beneficial to increase the nitrogen utilization of grape under subsurface drip irrigation

**DOI:** 10.3389/fpls.2023.1259516

**Published:** 2023-09-18

**Authors:** Dongdong Yao, Jianli Yang, Haifeng Jia, Yufan Zhou, Qi Lv, Xujiao Li, Huanhuan Zhang, Phillip Fesobi, Huaifeng Liu, Fengyun Zhao, Kun Yu

**Affiliations:** The Key Laboratory of Characteristics of Fruit and Vegetable Cultivation and Utilization of Germplasm Resources of the Xinjiang Production and Construction Corps, Shihezi University, Shihezi, Xinjiang, China

**Keywords:** fruit tree hole storage brick, grape, 15 N-urea, nitrogen absorption and distribution, subsurface drip irrigation

## Abstract

It is very important to promote plant growth and decrease the nitrogen leaching in soil, to improve nitrogen (N) utilization efficiency. In this experiment, we designed a new fertilization strategy, fruit tree hole storage brick (FTHSB) application under subsurface drip irrigation, to characterise the effects of FTHSB addition on N absorption and utilization in grapes. Three treatments were set in this study, including subsurface drip irrigation (CK) control, fruit tree hole storage brick A (T1) treatment, and fruit tree hole storage brick B (T2) treatment. Results showed that the pore number and size of FTHSB A were significantly higher than FTHSB B. Compared with CK, T1 and T2 treatments significantly increased the biomass of different organs of grape, N utilization and ^15^N content in the roots, stems and leaves, along with more prominent promotion at T1 treatment. When the soil depth was 15–30 cm, the FTHSB application significantly increased the soil ^15^N content. But when the soil depth was 30–45 cm, it reduced the soil ^15^N content greatly. T1 and T2 treatments obviously increased the activities of nitrite reductase (NR) and glutamine synthetase (GS) in grape leaves, also the urease activity(UR) in 30 cm of soil. Our findings suggest that FTHSB promoted plant N utilization by reducing N loss in soil and increasing the enzyme activity related to nitrogen metabolism. In addition, this study showed that FTHSB A application was more effective than FTHSB B in improving nitrogen utilization in grapes.

## Introduction

N is one of the essential elements for the growth of plants, and is also the main component of agricultural fertilizers (Jia et al., 2019). At present, the annual consumption of N fertilizer worldwide is as high as 1.5×10^8^ t (Cui et al., 2023), but the N utilization efficiency (NUE) is rather low. In most agricultural production, the NUE is only 30%–40% (Josep and Jordi, 2022; Wang et al., 2022b), which causes huge economic loss each year (Bodirsky et al., 2014). Farmers apply a large amount of N fertilizer in orchards to improve the yield and quality of fruits. The excessive application of N fertilizer has resulted in soil acidification (Tian and Niu, 2015; Yang, 2019), hardening, low nitrogen use efficiency (NUE) of plant (Xia et al., 2022). Furthermore, excessive application of N, causes a series of environmental problems such as poor soil quality(Gao et al., 2023; Zhang et al., 2023), eutrophication of surface water, excessive nitrate N in groundwater and air pollution (Qian et al., 2017; Erik, 2019). Therefore, how to apply fertilizer scientifically, reduce the use of N fertilizer and improve the N absorption and NUE of fruit trees has become a key issue for efficient and sustainable development of modern agriculture.

Subsurface drip irrigation as the main irrigation method in arid and semi-arid areas could improve fertilizer N utilization compared with traditional irrigation (Li et al., 2021; Han et al., 2022). Subsurface drip irrigation also effectively reduces the water loss in transportation, surface evaporation and deep leakage, contributing to remarkable water savings (Wang et al., 2021; Guo et al., 2022). The fertilization strategy adopting chemical fertilizer under drip irrigation has been widely used in the large-scale production of crops, especially that relying on a large amount of N fertilizer to increase the yield of crops, such as wheat, maize and cotton (Wang et al., 2022b; Dhayal et al., 2023). This mode of fertilization has increased the grain yield dramatically (Meshram et al., 2019). However, under this mode, more than half of the N fertilizer goes to the environment and causes a negative impact on the environment, which has drawn more and more attention around the world (Zou et al., 2020; Chen et al., 2023). Excessive application of N fertilizer not only leads to low fertilizer utilization efficiency and serious environmental problems, but also causes huge economic loss (Chen et al., 2018; Xu et al., 2020). As a result, the strategy of subsurface drip irrigation combined with chemical fertilizer is an effective measure to improve water use efficiency and promote plant growth, But there are still some shortcomings such as low NUE and serious environmental pollution, so we have to explore a new strategy to increase the utilization of N.

Compared with conventional materials, nano materials have small-size effect, interface effect and quantum size effect, and nano materials are prone to produce surface conjugation effect (Wang et al., 2020; Mohammad et al., 2021). Many studies have demonstrated that application of nano-fertilizer to plant could significantly increase the content of plant growth hormones, thus promoting plant growth (Lombi et al., 2019; Wang et al., 2022a). Nano materials also can stimulate root development and promote N uptake of crops (Alia et al., 2012). Additionally, using nano materials as the activator could increase the pore ratio and the specific surface area of biochar, and further improve the N adsorption (María et al., 2022). Vermiculite and montmorillonite are excellent natural nano materials which can preserve nutrients, hold and store water, and provide good air permeability (Nhung et al., 2019; Song et al., 2023). Nano materials have great water adsorption capacity, strong temperature buffering capacity and small volume weight, and have high cation exchange capacity and strong cation exchange adsorption capacity (Zhang et al., 2012; Zhang et al., 2020; Wang et al., 2023). However there are plenty of studies on natural nano materials (Fu et al., 2023; Naincy et al., 2023), but few reports focus on making nano materials and organic fertilizers into stable products to promote plant growth and fertilizer utilization.

The grape is cultivated worldwide due to its lucrative nature as a fruit crop that thrives in various climates (Grassi and De Lorenzis, 2021). Grapes have a high economic value because, in addition to being eaten fresh, they are used to produce juice and wine. Nevertheless, vineyard is to date one of the most erosive land uses in the world. As a result, vineyard soils are tend to have nitrogen leaching problems, poorly developed and thus prone to degradation. (Dylan et al., 2022). Currently there are fewer reports on whether nanomaterials can regulate the growth and development of grapevines (Zhong et al., 2023). Our laboratory has developed a new nano fertilizer. It is made by nano material and organic fertilizers. In this study, 2-year-old cutting seedlings of summer black grape were used as the experimental materials. Under subsurface drip irrigation, box planting precision control test and ^15^N isotope tracer method were adopted to show the effect of FTHSB on the growth of grape seedlings and the effect of ^15^N on the absorption and distribution of plants, to study application urea combined with different FTHSBs on soil nitrogen leaching and nitrogen absorption by grape trees, effects of nitrogen utilization and plant growth and development, to determine whether FTHSB can promote the growth of grape trees and screen the best combination of nano materials with organic fertilizer, to provide a theoretical basis for the application of nano materials in fruit trees.

## Materials and methods

### Test area

The experiment was conducted from September 2019 through December 2020 in the solar greenhouse of Shihezi Experimental Station, Xinjiang (45°19’N, 86°03’E). This area had a temperate continental climate, with an average annual temperature of 25.1°C and a rainfall of 125.0–207.7 mm. The solar greenhouse chamber had insulation layers and brick walls with no arch structures of cement column. The temperatures in the chamber were 36°C and 17°C in the day and night, respectively. The relative humidity was 75%–80%. The box planting test was adopted where 30 boxes (40 cm × 40 cm × 60 cm) were set with a spacing of 20 cm. Their sides and bottom were covered with black waterproof cloth to isolate them from the environment. The tested soil was sieved soil and yellow sand (sieved soil: yellow sand = 1:1). The screened soil was selected from the 0–20 cm depth soil in the vineyard of Shihezi University Experimental Station, and passed through a 40-mesh screen. Each cultivation box contained 128 kg soil. The basic physical and chemical properties of the tested soil were as follows: pH 7.56, organic matter 12.60 g kg^-1^, total nitrogen 0.43 g kg^-1^, available phosphorus 28.6 mg kg^-1^, available potassium 23 mg kg^-1^ and soil bulk density 1.40 g cm^-3^.

### Experiment design

**Figure 1:**
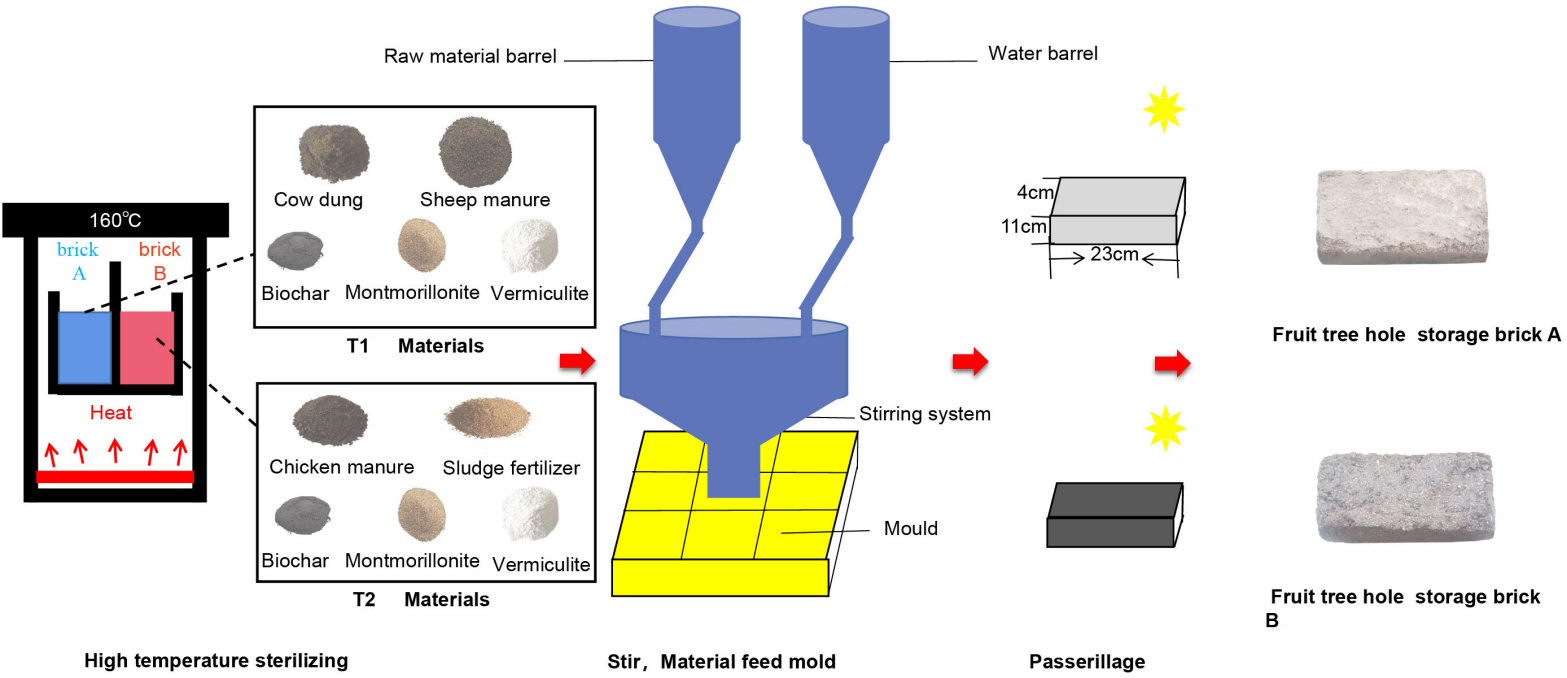
Production process of fruit tree hole storage brick.

Inspired by storing fertilizer and water in holes, our laboratory developed FTHSB. It is a nano-polymer brick-type product made of natural nano materials (montmorillonite and vermiculite), biochar, and organic fertilizers (cow dung, sheep dung and chicken dung), which are efficiently assembled by a customized mold. Nano materials were purchased from Anhui huabao biology co., ltd. Biochar samples were prepared by pyrolysis and carbonization of wheat stalks, provided by Henan Biochar Technology Engineering Laboratory and Henan Huinong Soil Conservation Co., Ltd. Air-dried sheep, cow and chicken manure were purchased from Hui Hui Fertilizer Co., Ltd., XinJiang, China. Firstly, the decomposed and fermented organic fertilizer is sterilized at high temperature, and the sterilized organic fertilizer and nano materials are screened by a 40-mesh sieve. Then, the materials are weighed according to the formula (FTHSB A: 300 g cow dung, 300 g sheep dung, 100 g vermiculite, 50 g montmorillonite, 10 g biochar; FTHSB B: 300 g chicken manure, 300 g oil residue, 100 g vermiculite, 50 g montmorillonite, 10 g biochar) and they are added into a blender. After fully stirred, they are evenly distributed to a mold with a length of 23 cm, a width of 11 cm and a height of 4 cm (add 1 L of water per 1520 g of materials). Then, the molds are coated with plastic wrap for fixing, and placed outdoors and naturally dried for 7 days (**Figure 1**).

**Figure 2:**
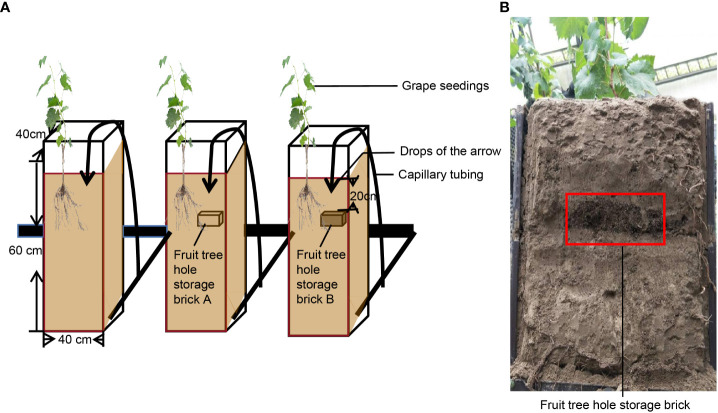
Experiments’ design of this study. **(A)** Pattern diagram of cultivation mode the three treatments. **(B)** picture of placement fruit tree hole storage brick in box.

There are three treatments in this experiment, Treatment I: subsurface drip irrigation (CK) control. Treatment II: fruit tree hole storage brick A (T1) treatment. Treatment III: fruit tree hole storage brick B (T2) treatment (**Figure 2A**). Each treatment is a single plot and has 10 repeating sets. In this study, two-year-old cutting seedlings of the’Summer Blake’ with 15–20 cm plant height, 4–5 functional leaves and a strong root system were selected. Grape seedlings were planted in the middle of the planting box on June 10. Before planting, different fruit tree storage bricks were applied 20 cm from the soil surface on one side of the planting box.(**Figure 2B**). After treatment, the drip irrigation belt was installed uniformly. The Ф50 mm PE pipe manufactured by Xinjiang Huili Water Saving Engineering Company was used as the main pipe, Ф20 mm PE pipe as the branch pipe, and Ф3/5 as the capillary pipe. A flow stabilizer (pressure compensated dripper, 1.5 L h^-1^) was connected between the capillary pipe and the branch pipe. Drop arrows were arranged at the depth of 5 cm from the soil surface just above the FTHSB, and subsurface drip irrigation was carried out until the soil water content reached 100%. After that, irrigation was carried out when the field water holding capacity was lower than 50%, and the irrigation amount per grape was 5 L. After the treatment with the FTHSB for 35 days, ^15^N-urea (2 g per grape) was dripped on one side directly above the FTHSB. Single factor randomized block design was adopted in the experiment, and other field cultivation and management processes were consistent.

### Plant biomass

After applying ^15^N-urea for 80 days, samples were taken. Five grape plants with basically the same growth were selected for destructive sampling in each treatment. All the soil of each layer was dug out separately, and roots were quickly collected and washed on a 100-mesh steel screen to minimize the loss of rhizomes. The roots, stems, and leaves of a whole plant were separated. Subsequently, they were rinsed three times with water, 1% hydrochloric acid, and deionized water, then the plant was dried 105°C for 30 min, baked at 80°C for 48 h and weighed. Later, the plants were crushed by a stainless steel grinder, screened by a 60-mesh sieve, packed in a plastic bag and stored in a dry place for later use.

### Transmission electron microscope

The nitrogen adsorption capacity of FTHSB A and FTHSB B were tested in lab-scale experiments by the following procedure. Briefly, two FTHSBs (A and B) were selected, and small cubes with a length, width and height of 1 cm were cut from their middle parts. 1 g ml^-1^ urea added to FTHSB A and FTHSB B. Then two bricks without urea and two bricks with urea were selected for the characterization of surface morphology and elemental distribution. The electron microscope image of FTHSB was scanned by SU8020 (JEOL, Japan) and the nitrogen content was determined by Horiba ex-350 energy spectrometer (JEOL, Japan).

### Nitrogen absorption and distribution in plants

On the 0–45 cm soil profile, the soil sample was taken every 15 cm, dried at 85°C to constant weight, and ground through a 60-mesh sieve. After the amount of biomass was measured, the screened samples of plant organs and screened soil samples of different soil layers were taken to determine the nitrogen content and nitrogen abundance. ^15^N abundance was measured by ZHT-03 mass spectrometer (Beijing analytical instrument factory).

Plant ^15^N calculation formula according to the method described by (Sha et al., 2021)


(1) $$\begin{array}{c} \text{Plant Ndf}f\;\left(\%\right)\;=\;\left({\text{abundance of }}^{15}{\text{N in plant-natural abundance of }}^{15}\text{N}\right)/({\text{abundance of }}^{15}\\ {\text{N in fertilizer-natural abundance of }}^{15}\text{N})\;\times \;100\% \end{array}$$

(2) $$\begin{array}{c} {\;}^{15}\text{N distribution rate }\left(\%\right)\;={\;}^{15}\text{N }\mathrm{absorbed}\;\mathrm{by}\;\mathrm{each}\;\mathrm{organ}\;\mathrm{from}\;\mathrm{fertilizer}\;\left(\text{g}\right)/{\text{total }}^{15}\\ \text{N absorbed by plant from fertilizer }\left(\text{g}\right)\;\times \;100\% \end{array}$$

(3) $$\begin{array}{c} {\;}^{15}\text{N utilization efficiency }\left(\%\right)\\ \;=\;[\text{Plant Ndff }\times \text{ total nitrogen in organs }\left(\text{g}\right)]/\text{fertilizer amount }\left(\text{g}\right)\times 100\% \end{array}$$

### Residual nitrogen in different soil layers

Samples were taken 80 days after ^15^N-urea was dripped, and five grape plants with basically the same growth were selected for each treatment. In the depth of 45 cm soil profile, samples were taken every 15 cm at one side of the FTHSB by quartering method, and soil sample of the same layer were mixed and put into aluminum boxes. After drying in an oven at 85°C to constant weight, the soil nitrogen content and ^15^N content were determined.

Soil ^15^N calculation formula according to the method described by (Dai et al., 2023)


(4) $$\begin{array}{c} \text{Soil Ndff }\left(\%\right)\;=\;\left({\text{abundance of }}^{15}{\text{N soil-natural abundance of }}^{15}\text{N}\right)/({\;}^{15}\\ \text{N Abundance natural abundance of the fertilizer})\;\times \;100\% \end{array}$$

(5) $${\;}^{15}\text{N content in soil = Total nitrogen in soil }\times \text{Soil Ndff}$$

### Nitrogen metabolism enzyme activity

The activity of nitrate reductase (NR) was measured, and samples were taken 80 days after ^15^N-urea was dripped. Five grape plants with basically the same growth were selected for each treatment, and the functional leaves were measured. The activity of nitrate reductase was measured according to (Marium et al., 2023). For this, 200 mg plant sample was extracted using 100 mM phosphate buffer with a pH of 7.5, 30 mM KNO_3_, and 5% propanol. The tubes were kept in a water bath maintained at 100°C for 5 min. 10 mL of colour reagent [N-(1-naphthyl)-ethylenediamine hydrochloride] and 0.02% Griess reagent [N-(1-naphthyl)-ethylenediamine hydrochloride] were supplemented to the medium to determine the NO_2_-N produced. To calibrate the colour reaction, a NO_3_ stock solution containing 25 M potassium nitrite (KNO_3_) was used. The absorbance of the supernatants was measured at 540 nm by using a UV/Visible spectrophotometer (Specord 200, Analytik Jena, Germany).

The activity of Glutaminase activity (EC3.5.1.2) was assayed using a glutaminase kit (Beijing Solarbio Science & Technology Co., Ltd., Beijing, China) with the specification of 50 tubes/24 samples. The method was visible spectrophotometry, and 1 g of leave-catalyzed glutamine production of 1 µmol L^−1^ ammonia per day at 37°CC was defined as one enzyme activity unit (U g^−1^) (Liu et al., 2021).

Urease activity (EC3.5.1.5) was assayed by colorimetric analysis of sodium phenate-sodium hypochlorite (Van et al., 2017)

### Data processing

The experiment data were counted by Excel 2010, analyzed by SPSS 16.0 for variance, by using one-way factorial analysis of variance (ANOVA). Origin 2018 was used for figure drawing. In all cases, differences were considered significant at a probability level of P ≤ 0.05. Furthermore, correlation analyses using R studio 4.0.3 (corrplot R 4.0.3).

## Results

### Nitrogen absorption capacity of FTHSB with different compositions

**Figure 3:**
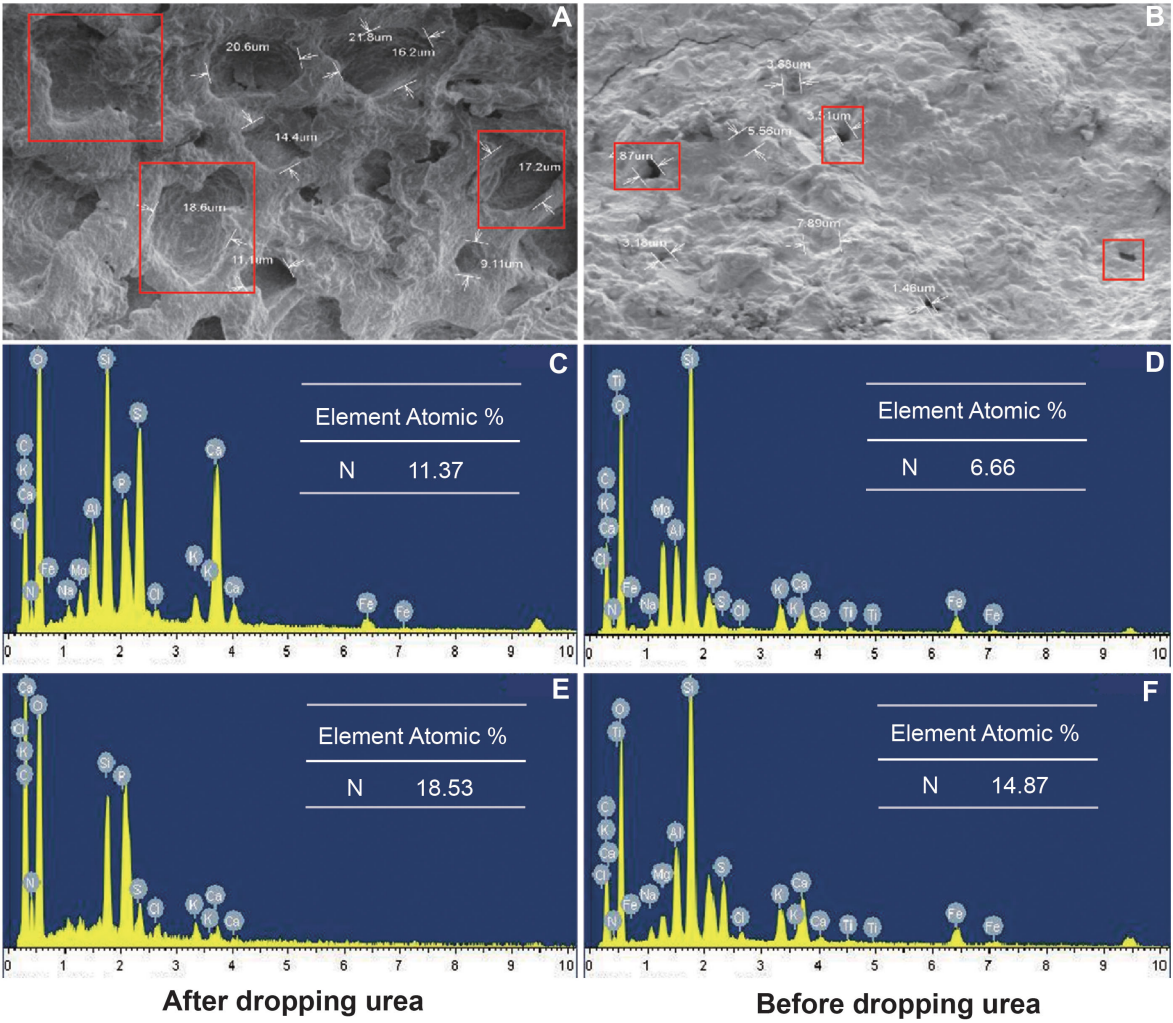
Different FTHSB nitrogen content before and after urea drip application. **(A, B)** Scanning electron micrographs exhibit different porosity for these two types of bricks with pore sizes, Red frame on pictures show the different pore sizes in FTHSB A and FTHSB **(B, C)** nitrogen content in FTHSB B after dripping urea. **(D)** nitrogen content in FTHSB B before dripping urea. **(E)** nitrogen content in FTHSB A after dripping urea. **(F)** nitrogen content in FTHSB A before dripping urea.

FTHSBs were analyzed with TEM-EDS to ascertain the surface morphology and elemental components (**Figures 3A-F**). Both FTHSB A and FTHSB B showed the uniform morphology, and multi-micro-pores structurethe shape was also observed. Compared to FTHSB A, FTHSB B presented the larger micro-pores. **Figure 3A** shows that T1 has more micro-pores and the largest pore has a diameter of 21.8 μm. **Figure 3B** shows that T2 has fewer small poles and the maximum pore diameter is 4.87 μm. TEM-EDS was used to measure the N content of FTHSB before and after dripping urea. The results are shown in **Figures 3C, D**. Before dripping urea, the N content of T1 was 123.27% higher than T2 (expressed as the percentage of atomic molar weight). After dripping urea, the N content of T1 was 62.97% higher than T2 (**Figures 3E, F**).

### The biomass of plant organs

According to **Table 1**, The dry matter quantity of roots, stems and leaves of plants under T1 and T2 treatments were also significantly higher than CK (*P*< 0.05), among which the dry matter quantity of roots was the highest, 68.95% and 56.55% higher than those under CK, respectively. Among the three treatments, the dry matter quantity of each organ was in the order of root > leaf > stem. Under T1 treatment, the dry matter quantity of each organ reached the maximum, and the dry matter quantity of root, stem and leaf were 7.92%, 14.03%, 12.67% higher than those of T2 treatment, respectively. These results indicated that compared to CK, T1 and T2 treatments had a significant stimulating effect on plant growth, and the effect of T1 treatment was greater than that of T2 treatment.

**Table 1:** Effect of different FTHSB Treatments on biomass of different organs of grape seedlings.

Treatment	Root (g)	Stem (g)	Leaf (g)
CK	27.50 ± 0.01^b^	14.67 ± 2.97^c^	18.46 ± 1.23^b^
T1	46.46 ± 0.07^a^	21.28 ± 0.89^a^	25.17 ± 0.85^a^
T2	43.05 ± 0.06^a^	18.82 ± 0.94^b^	22.34 ± 2.29^a^

Data are the mean standard error (n = 3). a, b indicate the standard error of the mean. Values followed by different letters within the same column indicate significant differences at P< 0.05. CK, conventional subsurface drip irrigation treatment; T1, fruit tree hole storage brick A treatment; T2, fruit tree hole storage brick B treatment.

### Ndff value and ^15^N distribution rate of plant organs

**Figure 4:**
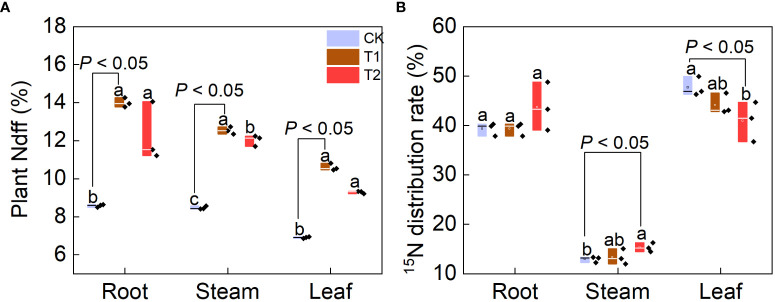
Effect of FTHSB on Ndff **(A)** and ^15^N **(B)** distribution of grape root, stem and leaf. Data are the mean standard error (n = 3). Different letters indicate significant differences by Duncan’s test among treatments (*P*< 0.05). CK, conventional subsurface drip irrigation treatment; T1, fruit tree hole storage brick A treatment; T2, fruit tree hole storage brick B treatment. Within a column section, values that differ significantly (*P* < 0.05) are followed by different lower case letters, as determined by one-way analysis of variance (ANOVA) incorporating Tukey’s HSD test for pair-wise comparisons between means.

The Ndff value of an organ refers to the contribution rate of ^15^N absorbed and distributed from ^15^N fertilizer to the total N content of the organ, which reflects the ability of plant organs to absorb and regulate ^15^N in the fertilizer (Quan et al., 2021). All organs of grape plants under the three treatments had the highest Ndff value in roots and the lowest in leaves, which implies that the roots has the strongest ability to transport ^15^N at the grape seedling stage, higher than leaves and stems (**Figure 4A**). Among the three treatments, Ndff values of roots, stems and leaves of plants under T1 and T2 treatments were significantly higher than those under CK (*P*< 0.05). Among them, the Ndff value of each organ under T1 treatment was the highest, and the Ndff values of roots, stems and leaves were 14%, 12.53% and 10.61%, respectively.

The percentage of ^15^N in each organ to the total amount of ^15^N in the whole plant reflects the distribution of fertilizer N in the tree and the law of its migration in each organ (Xiao et al., 2019). The distribution rate of ^15^N in each organ of the grape seedlings under T1 or CK treatment was leaf > root > stem, and under T2 treatment, the distribution rate was root > leaf > stem (**Figure 4B**). It means that ^15^N under the three treatments was mainly stored in the roots and leaves. The distribution rate of ^15^N in roots and stems was T2 > T1 > CK, but the highest distribution rate of ^15^N in leaves was CK treatment.

### Total nitrogen content, ^15^N absorption and ^15^N utilization efficiency of grape plants

The effects of different treatments on the total N content, the absorptive amount and utilization efficiency of ^15^N of grape seedlings were shown in **Table 2**. Compared those treated with CK, the total N content of plants treated with T1 and T2 increased significantly (*P*< 0.05). The results showed that T1 and T2 significantly increased the absorptive amount and utilization efficiency of ^15^N of grape seedlings [T1 > T2 > CK (*P*< 0.05)]. The ^15^N absorptive amount of grape seedlings under T1 treatment was 128.57% and 6.67% higher than those under CK and T2 treatments respectively. The ^15^N utilization efficiency of grape seedings was 111.41% and 12.28% higher than those under CK and T2 respectively.

**Table 2:** Total nitrogen and ^15^N absorption and utilization rate of grape plants by FTHSB.

Treatment	Plant total nitrogen content (g)	^15^N absorption (g)	^15^N utilization rate (%)
CK	0.96 ± 0.07^b^	0.07 ± 0.01^b^	3.33 ± 0.25^b^
T1	1.33 ± 0.09^a^	0.16 ± 0.01^a^	7.04 ± 0.55^a^
T2	1.35 ± 0.06^a^	0.15 ± 0.01^a^	6.27 ± 0.24^a^

Data are the mean standard error (n = 3). Different letters indicate significant differences by Duncan’s test among treatments (P< 0.05). CK, conventional subsurface drip irrigation treatment; T1, fruit tree hole storage brick A treatment; T2, fruit tree hole storage brick B treatment.

### The activity of nitrogen metabolic enzymes

**Figure 5:**
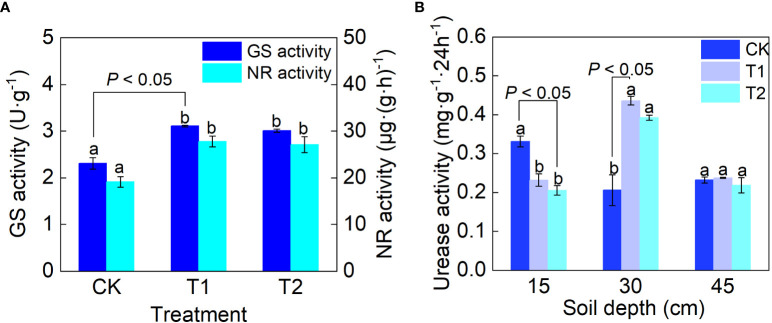
Effect of FTHSB on **(A)** NR and GS activities of plant leaves and UR **(B)** activity of soil. Data are the mean standard error (n = 3). Different letters indicate significant differences by Duncan’s test among treatments (*P*< 0.05). CK, conventional subsurface drip irrigation treatment; T1, fruit tree hole storage brick A treatment; T2, fruit tree hole storage brick B treatment.

Nitrate reductase (NR) and glutamine synthetase (GS) are the key enzymes in nitrogen metabolism of plants (Wang et al., 2022b). Compared with CK, T1 and T2 treatments increased the activities of NR and GS significantly (**Figures 5A, B**). Among the three treatments, the NR activities in leaves under T1 and T2 treatments were 45.42% and 41.81% higher than that under CK, and the GS activities in leaves were 34.63% and 30.31% higher with significant differences (*P<* 0.05). Among all treatments, T1 treatment had the most significant effect on promoting NR and GS activities in leaves of grape seedlings, with NR and GS activities of 3.11 U g^-1^ and 27.79 μg g^-1^ h^-1^, respectively.

The urease (UR) activity of soil from 15 cm soil layer under CK treatment was significantly higher than that under T1 and T2 treatment (*P*< 0.05) (**Figure 5C**). T1 and T2 treatments significantly improved the activity of urease in 30 cm soil layer, increased by 110% and 89.8% respectively compared with CK (*P*< 0.05). Among the treatments, T1 had the most obvious effect on promoting UR activity in 30 cm soil layer.

### ^15^N content in different soil layers

**Figure 6:**
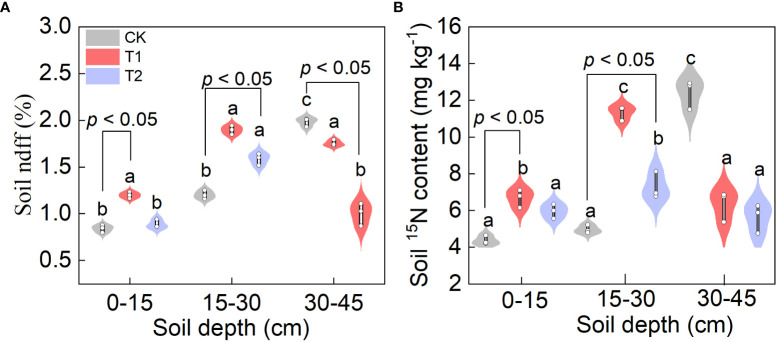
Effect of FTHSB on Ndff **(A)** and ^15^N content **(B)** in different soil layers. Data are the mean standard error (n = 3). Different letters indicate significant differences by Duncan’s test among treatments (*P*< 0.05). CK, conventional subsurface drip irrigation treatment; T1, fruit tree hole storage brick A treatment; T2, fruit tree hole storage brick B treatment.

The Ndff under CK increased with the increase of soil depth. Under T1 and T2 treatments, Ndff first decreased and then increased with the deepening of soil depth **(** **Figure 6A** **)**. At the depth of 15 cm, the Ndff value under T1 was 42.86% and 37.93% significantly higher than CK and T2, respectively (*P*< 0.05). At the soil layers of 30 cm depth, the Ndff values under T1 and T2 treatments were significantly higher than those under CK treatment (*P<* 0.05). At the soil layers of 45 cm depth, the Ndff values under CK were significantly higher than those under T1 and T2 (*P<* 0.05).

With the increase of soil depth, the soil ^15^N content under CK treatment increased gradually, while the soil ^15^N content under T1 and T2 treatments increased first and then decreased (**Figure 6B** **)**. At the depth of 15 cm and 30 cm, the contents of ^15^N in soil treated with T1 and T2 treatments were significantly higher than that under CK treatment and the differences were significant (*P<* 0.05). The maximum value was reached at 30 cm, the ^15^N in soil under T1 and T2 treatments were 128.11% and 46.72% higher than CK treatment. At the depth of 45 cm, the ^15^N content under CK treatment was significantly higher than those under T1 and T2 treatments (*P<* 0.05).

### Correlation between the utilization and distribution of N, the biomass of plants and the activity of N metabolic enzymes

**Figure 7:**
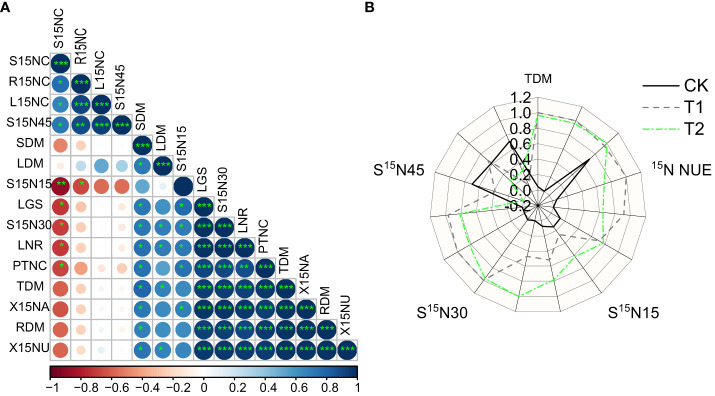
Correlation analysis of change **(A)** of root dry matter (RDM), steam dry matter (SDM), leaf dry matter (LDM), total dry matter (TDM) and root NDFF (RNDFF), steam (SNDFF), leaf (LNDFF), root ^15^N content (R15NC), steam ^15^N content (S15NC), leaf ^15^N content (L15NC), plant ^15^N utilization (P15NU), plant ^15^N allocation (P15NA), plant total nitrogen content (PTNC), soil NDFF 15, 30, 45 (SNDFF15, SNDFF30, SNDFF45), 15, 30, 45 cm soil ^15^N content (S15N15, S15N30, S15N45), leaf NR activity (LNR), leaf GS activity (LGS). *significant at the 5% probability level; **ignificant at the 1% probability level; ***significant at the 0.1% probability level. Synergies and potential tradeoffs associated with different treatments **(B)**, TDM of total plant dry matter, utilization rate of plant ^15^N (P15NU), and ^15^N content (S^15^N15, S^15^N30, S^15^N45) in soil at 15, 30 and 45 cm.

Plant utilization of 15N was significantly and positively correlated with the amount of dry matter quantity per plant organ and total biomass (*P*< 0.05), extremely significantly positively correlated with the ^15^N content in 15–30 cm soil layer (*P<* 0.01) (**Figure 7A** **)**. But the utilization efficiency of ^15^N in plants was negatively correlated with the ^15^N content in 30–45 cm soil layer. The activities of NR and GS in leaves were positively correlated with the ^15^N content in 30 cm soil layer and negatively correlated with the ^15^N content in 45 cm soil layer.

Compared with CK treatment, T1 and T2 treatments increased the dry matter quantity, NUE and ^15^N content in 30 cm soil, and decreased the ^15^N content in 45 cm soil **(** **Figure 7B** **)**. Under T1 treatment, the total biomass of grape seedlings, ^15^N content in 30 cm soil layer and ^15^N utilization efficiency all reached the maximum value. This indicated that adopting FTHSB under the subsurface drip irrigation could promote plant growth, improve plant nitrogen utilization and effectively reduce nitrogen loss. T1 was more effective than T2 in promoting plant growth and nitrogen absorption and utilization.

## Discussion

### Adsorbability of different FTHSBs

The effects of nanomaterials on plants and the environment may largely determine the direction of their application and their potential in agricultural production (Ren et al., 2021). Many studies have confirmed that nano materials have a positive effect on improving NUE (Mukherjee et al., 2016). In this study, nitrogen content of FTHSB A was higher than that of FTHSB B, after drip application of urea. A popular explanation behind the difference in the ability to adsorb N between T1 and T2 could be as follows: the surface area and pore volume of T1 was larger than that of T2, the ion exchange process will lead to increase the surface and pore structure of montmorillonite and other nanomaterials and these changes will affect the adsorption characteristics of T1 and T2 (Huang et al., 2004). Through electron microscopy, we could also see that the number and size of pores in T1 were significantly more than those in T2, which was also the reason for the stronger N adsorption capacity of FTHSB A than FTHSB B. In addition, nano materials not only can enhance plant N adsportion but also can improve the retention and migration capacity of N in soil, thus directly or indirectly promoting plant root growth (Fabio, 2019; Zhu et al., 2019; Pisa et al., 2020).

### Effect of FTHSB on plants growth and nitrogen uptake and utilization

A large number of studies showed that adding nano materials can greatly promote the growth of plants and increase the biomass of plants (Brandl et al., 2015; Sandeep et al., 2018). Nanomaterials as a new type of nano-phosphate fertilizer, could significantly promote plant growth (Ahmed et al., 2022). In this study we found that adopting FTHSB could significantly increase the biomass of grape seedlings compared with the control. The biomass of grape seedlings reached the maximum value under T1 treatment, with an increase of 36.34% compared with CK. Under T1 and T2 treatments, the biomass in roots and leaves was significantly higher than those under CK. The application of FTHSB boosted grape saplings growth and development of roots, the growth and development of roots directly affects the absorption of soil nutrients and soil water by roots and the growth and development of trees. (Cao et al., 2019; Zhang et al., 2021). Therefore, it is a feasible measure to promote the growth of grapes by applying FTHSB on the basis of subsurface drip irrigation in the greenhouse chamber.

After application of chemical fertilizers by spreading, soil available nitrogen concentration will rapidly increase in a short period of time. During this period, rainfall or irrigation will result in nitrogen loss (Jamal et al., 2023). Although drip fertilization can directly transport nitrogen fertilizer to roots of plants, it can also cause serious problems including N leakage, leaching and volatilization. Nano materials could improve soil physical and chemical properties, effectively adsorb nitrogen in soil, reduce N loss, and significantly improve the plant NUE and the soil N residual rate (Wang et al., 2018; Sandra et al., 2022). In this experiment, we used grape seedlings as test materials, and analyzed the effects of applying FTHSB to grape seedlings on the utilization and distribution of ^15^N under subsurface drip irrigation in greenhouse chambers. The results showed that compared with CK, T1 and T2 treatments significantly increased the NUE of grape seedlings. The reason might be that the application of FTHSB effectively absorbed N, increased N content in soil, and effectively reduced N leaching. The FTHSB also promotes the growth of the root system, and then promotes the absorption of N by grape seedlings. Previous studies have demonstrated that adding biochar increased the crop yield, promoted the absorption and utilization of N, reduced the N content in stems and increased the N content in grains (Qian et al., 2017). In this experiment, T1 and T2 treatments increased the distribution of ^15^N in roots and stems but decreased the distribution in leaves. This is inconsistent with previous studies, which may be related to the interaction of various materials and its unique structure of the FTHSB. N was absorbed by the grape root system and transferred to the shoot in the plant, application of FTHSB affected the uptake of N, which in turn affects N transport in the plant and in various organs allocation.

**Figure 8:**
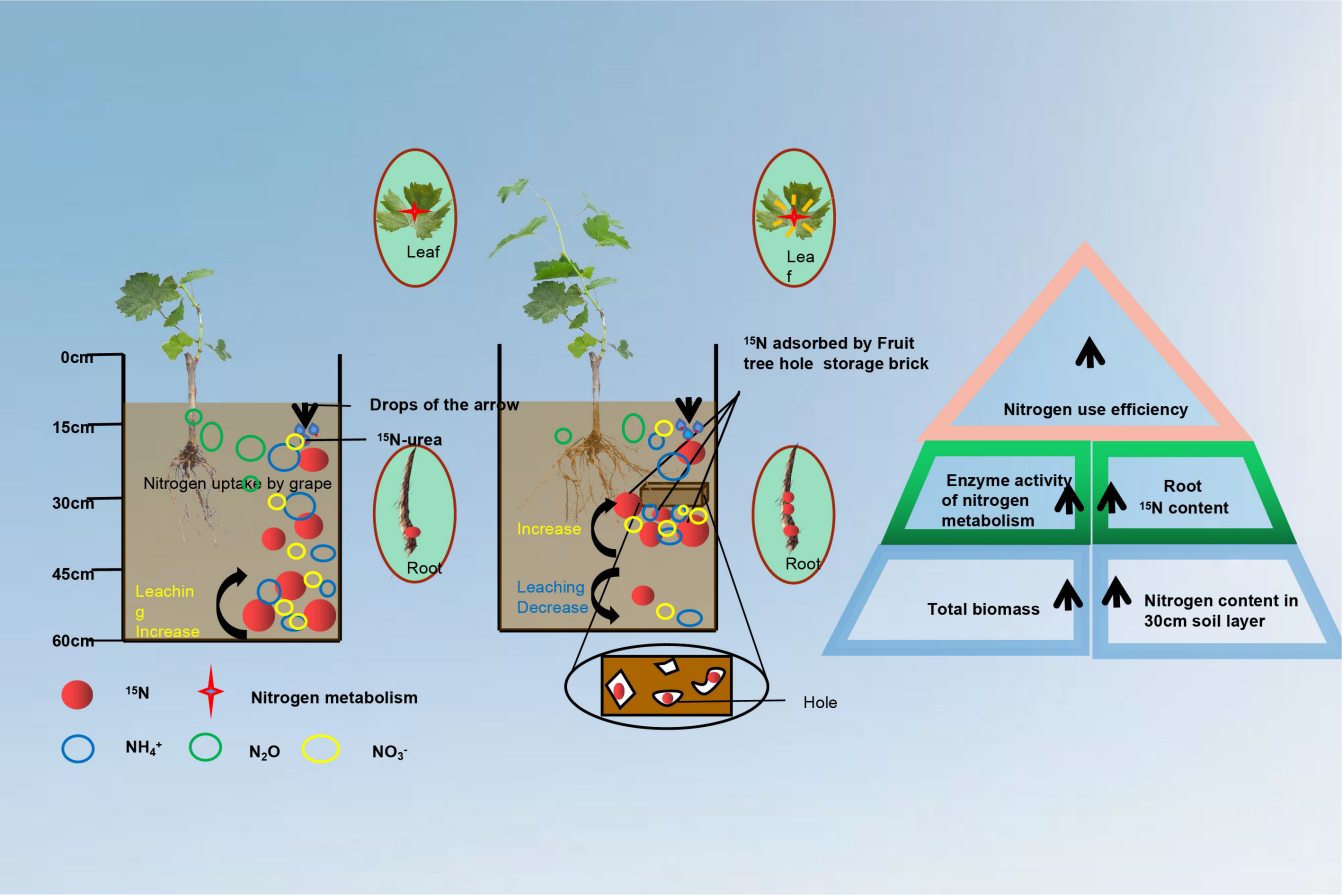
Application of FTHSB to grape seedling growth and nitrogen use efficiency strategy diagram.

NR, GS and soil UR are the key enzymes for plants to transform and utilize nitrogen, and the activity of these enzymes reflects the N utilization ability (Wu et al., 2020). In this study, the NR activities in leaves treated with T1 and T2 were 45.4% and 41.8% higher than that under CK, respectively. The differences were significant. GS activity was the highest in T1 treatment and the lowest in CK treatment which was significantly lower than that in T1 and T2 treatment. The soil UR activity plays a great significance role in transformation of relatively stable organic N into inorganic N in soil, which can improve the situation of soil providing N nutrients to plants and enhance the N supply capacity of soil (Zhao et al., 2021). In this study, the soil UR activity from the 30 cm soil layer under T1 and T2 treatment was significantly higher than that under CK. These results showed that FTHSB significantly increased NR and GS activities in plant leaves and the soil UR activity in 30 cm soil layer (**Figure 8**). Correlation analysis showed that NR and GS activities were positively correlated with the total nitrogen content, ^15^N absorptive amount of the plant, ^15^N content in 30 cm soil, ^15^N utilization efficiency, plant dry matter quantity and with ^15^N content in 15 cm soil, but NR and GS activities were negatively with ^15^N content in 45 cm soil. This indicated that the application of FTHSB could significantly improve NR and GS activities, which increased nitrogen absorption and utilization capacity of plants, and reduced the residual ^15^N in 30–45 cm soil. In addition, the FTHSB is an organic brick with high surface energy which is made of vermiculite, montmorillonite, biochar and other small-sized materials. It has good performance in improving the NUE of plants, promoting the growth, and affecting the N metabolic process, which may be due to its absorption of NH_4_^+^, NO_3_^-^ and small molecules of urea. The influence of nano materials on specific N metabolic process of plants needs to be further explored.

### Effect of FTHSB on fertilizer N residues in the soil

Soil is the main place for the storage and transformation of nutrients such as N and phosphorus (Turner and Haygarth, 2001; Pellegrini et al., 2018). After general chemical fertilizer was applied to an orchard, it came into direct contact with the soil and dissolved quickly, which increased the nitrogen concentration in the soil (Zeng et al., 2021). As a result, N loss occurred in a very short period of time. However, the nutrients in FTHSB were released slowly through the microholes in the brick, which kept the nitrogen concentration in the soil at a stable level (Ren et al., 2022). Adding biochar could improve the N fertilizer utilization efficiency, which was mainly due to the fact that biochar improves the water retention and cation exchange capacity of fluvo-aquic soil, thus increasing the nitrogen uptake by aboveground parts (Nikolas et al., 2017; Li et al., 2020). In addition, natural nanomaterials vermiculite and montmorillonite could also improve the retention of nitrogen and reduce the loss in soil, thus improving the NUE of plants. Previous developed a new type of slow-release fertilizer (SRFs) with a kind of bio-based waterborne polymeric coating, compared with adding urea only, adding slow-release fertilizer increased the NUE by 68.3% (Mehri et al., 2021). In this study, the content of ^15^N in soil increased first and then decreased after T1 and T2 treatments where FTHSBS were applied. T1 and T2 treatments prevented the migration of nitrogen to deep soil and increased the N residue in the middle soil, especially T1 treatment. This is mainly because the adhesion of nanomaterials makes the surface of cow manure and sheep manure rough and increases the specific surface area. It may also because the water vapor generated in the combination process improves the pore structure and increases the specific surface area, and the fertilizer ^15^N was largely adsorbed in the soil after application of FTHSB. Biochar, which has a high surface area and pore volume, generally has a stronger physisorption capacity in terms of N, with the hydrated asymmetric N ions becoming physically trapped within the biochar pores when solution flows into the biochar particles (Pratiwi et al., 2016; Joseph et al., 2018; Sanford et al., 2019). In this study, applying FTHSB is beneficial to storage surplus N retention in the soil, reduce N volatilization and leaching, this may be related to nano-materials combined with organic fertilizer resulting in an increase in surface area due to the greater number of organo-mineral (plaques) layers loaded onto its external and internal surfaces, more positively charged minerals and cationic salts on the surface of FTHSB subsequently form on both the internal and external surfaces of the FTHSB, leaving its pores blocked and trapping N within, thereby increasing the adsorption and retention capacity of ^15^N (**Figure 8**).

## Conclusion

Application of FTHSB under subsurface drip irrigation can promote the growth of grape seedlings, decrease the leaching loss of N to the deep soil, increased activity of nitrogen metabolizing enzymes and it also improves the utilization efficiency of N. Among all treatments, FTHSB A fertilizer had the most significant promotion effect. These results indicate that FTHSB fertilizer has a huge potential for improving NUE and economic benefits of orchards under subsurface drip irrigation, and it also serves as a theoretical basis for promoting fruit production in orchards.

## Data availability statement

The original contributions presented in the study are included in the article/supplementary material. Further inquiries can be directed to the corresponding authors.

## Author contributions

DY: Data curation, Formal Analysis, Investigation, Methodology, Software, Writing – original draft, Writing – review & editing. JY: Data curation, Formal Analysis, Investigation, Methodology, Software, Writing – original draft. HJ: Supervision, Writing – review & editing. YZ: Writing – review & editing. QL: Writing – review & editing. XL: Writing – review & editing. HZ: Writing – review & editing. PF: Writing – review & editing. HL: Writing – review & editing. FZ: Writing – review & editing. KY: Conceptualization, Funding acquisition, Project administration, Resources, Supervision, Writing – original draft.
